# 2,3-Dibromo-3-(2-bromo­phen­yl)-1-(3-phenyl­sydnon-4-yl)propan-1-one

**DOI:** 10.1107/S1600536810044776

**Published:** 2010-11-06

**Authors:** Hoong-Kun Fun, Madhukar Hemamalini, Nithinchandra, Balakrishna Kalluraya

**Affiliations:** a X-ray Crystallography Unit, School of Physics, Universiti Sains Malaysia, 11800 USM, Penang, Malaysia; b Department of Studies in Chemistry, Mangalore University, Mangalagangotri, Mangalore 574 199, India

## Abstract

In the title compound [systematic name: 2,3-dibromo-3-(2-bromo­phen­yl)-1-(5-oxido-3-phenyl-1,2,3-oxadiazol-3-ium-4-yl)propan-1-one], C_17_H_11_Br_3_N_2_O_3_, the oxadiazole ring is essentially planar, with a maximum deviation of 0.003 (1) Å. The –CHBr–CHBr– chain and bromo­phenyl ring are disordered over two sets of sites with a refined occupany ratio of 0.756 (5):0.244 (5). The central oxadiazole ring makes dihedral angles of 54.07 (11) and 13.76 (18)° with the attached phenyl and the major component of the bromo-substituted benzene rings, respectively. The dihedral angle between the major and minor components of the bromo­phenyl rings is 13.4 (5)°. In the crystal structure, mol­ecules are connected by C—H⋯O hydrogen bonds, forming [010] ribbons.

## Related literature

For applications of sydnones, see: Rai *et al.* (2008 ▶); Jyothi *et al.* (2008 ▶). For details of chalcones, see: Rai *et al.* (2007 ▶). For the stability of the temperature controller used in the data collection, see: Cosier & Glazer (1986 ▶).
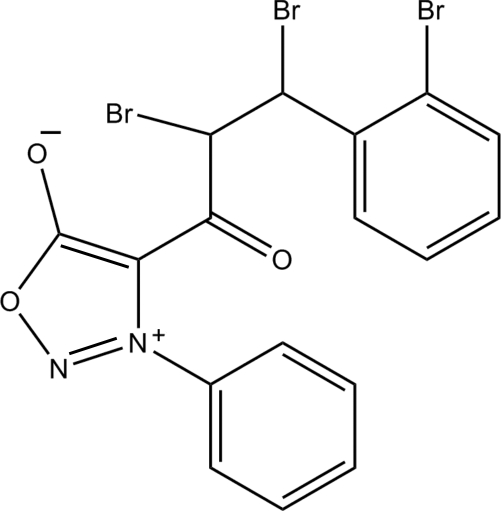


## Experimental

### 

#### Crystal data

C_17_H_11_Br_3_N_2_O_3_
*M* *_r_* = 531.01
Monoclinic, 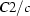

*a* = 29.0105 (16) Å
*b* = 7.2271 (4) Å
*c* = 17.7209 (9) Å
β = 102.591 (2)°
*V* = 3626.0 (3) Å^3^
*Z* = 8
Mo *K*α radiation
μ = 6.69 mm^−1^
*T* = 100 K
0.41 × 0.17 × 0.12 mm

#### Data collection

Bruker APEXII DUO CCD diffractometer
Absorption correction: multi-scan (*SADABS*; Bruker, 2009 ▶) *T*_min_ = 0.169, *T*_max_ = 0.503
44623 measured reflections
6547 independent reflections
5223 reflections with *I* > 2σ(*I*)
*R*_int_ = 0.040

#### Refinement

*R*[*F* ^2^ > 2σ(*F* ^2^)] = 0.027
*wR*(*F* ^2^) = 0.099
*S* = 1.04
6547 reflections
277 parameters
3 restraints
H-atom parameters constrained
Δρ_max_ = 0.71 e Å^−3^
Δρ_min_ = −0.93 e Å^−3^

### 

Data collection: *APEX2* (Bruker, 2009 ▶); cell refinement: *SAINT* (Bruker, 2009 ▶); data reduction: *SAINT*; program(s) used to solve structure: *SHELXTL* (Sheldrick, 2008 ▶); program(s) used to refine structure: *SHELXTL*; molecular graphics: *SHELXTL*; software used to prepare material for publication: *SHELXTL* and *PLATON* (Spek, 2009 ▶).

## Supplementary Material

Crystal structure: contains datablocks global, I. DOI: 10.1107/S1600536810044776/hb5718sup1.cif

Structure factors: contains datablocks I. DOI: 10.1107/S1600536810044776/hb5718Isup2.hkl

Additional supplementary materials:  crystallographic information; 3D view; checkCIF report

## Figures and Tables

**Table 1:** Hydrogen-bond geometry (Å, °)

*D*—H⋯*A*	*D*—H	H⋯*A*	*D*⋯*A*	*D*—H⋯*A*
C5—H5*A*⋯O3^i^	0.93	2.46	3.129 (2)	129
C10*A*—H10*A*⋯O2	0.98	2.30	3.060 (3)	133
C11*A*—H11*B*⋯O2^ii^	0.98	2.47	3.231 (3)	134
C17*A*—H17*A*⋯O2^ii^	0.93	2.48	3.315 (5)	149

Symmetry codes: (i) 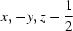; (ii) 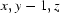.

**Fig. 1.**
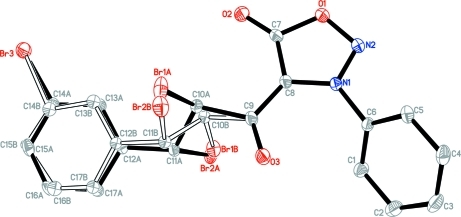
The asymmetric unit of the title compound, showing 30% probability displacement ellipsoids (H atoms are omitted for clarity). Open bonds represent the minor disorder component.

**Fig. 2.**
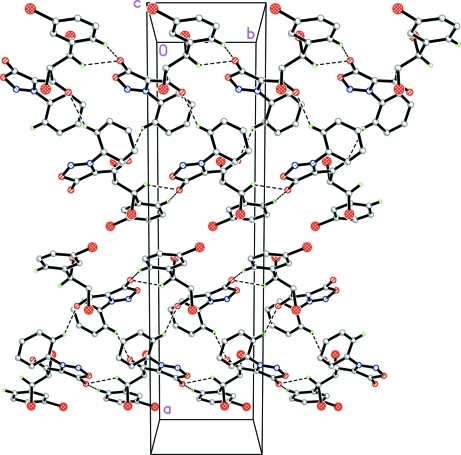
The packing of the title compound, showing hydrogen-bonded chains down the b axis.

## supplementary crystallographic information

### Comment

Sydnones constitute a well-defined class of mesoionic compounds that contain the 1,2,3-oxadiazole ring system. The study of sydnones still remains a field of interest because of their electronic structure and also because of the varied types of biological activities (Rai *et al.*, 2008). Recently, sydnone derivatives were found to exhibit promising antimicrobial properties (Jyothi *et al.*, 2008). Chalcones were obtained by the base-catalyzed condensation of 4-acetyl-3-aryl sydnones with aromatic aldehydes in alcoholic medium employing sodium hydroxide as catalyst at 0–50°C. Bromination of chalcones with bromine in glacial acetic acid afforded dibromo chalcones (Rai *et al.*, 2007).

The molecular structure of the title compound is shown in Fig. 1. The oxadiazole (N1/N2/O1/C7/C8) ring is essentially planar, with a maximum deviation of 0.003 (1) Å for atom N1. The dibromo-substituted bromophenyl ring is disordered over two sites with a refined occupany ratio of 0.756 (5):0.244 (5). The central oxadiazole (N1/N2/O1/C7/C8) ring makes dihedral angles of 54.07 (11)° and 13.76 (18)° with the attached phenyl (C1–C6) and the bromo-substituted phenyl (C12A–C17A) rings, respectively. The dihedral angle between the major component (C12A–C17A) and the minor component (C12B–C17B) bromophenyl rings is 13.4 (5)°.

In the crystal, (Fig. 2), the molecules are connected by intermolecular C5—H5A···O3, C11A—H11B···O2 and C17A—H17A···O2 (Table 1) hydrogen bonds into ribbons along the *b* axis.

### Experimental

1-(3^1^-Phenylsydnon-4^1^-yl)-3-(*o*-bromophenyl)-propen-1-one (0.01 mol) was dissolved in glacial acetic acid (25–30 ml) by gentle warming. A solution of bromine in glacial acetic acid (30% w/v) was added to it with constant stirring till the yellow colour of the bromine persisted. The reaction mixture was stirred at room temperature for 1–2 hours. The solid which separated was filtered, washed with methanol and dried. It was then recrystallized from ethanol. Colourless blocks of (I) were obtained from 1:2 mixtures of DMF and ethanol by slow evaporation.

### Refinement

All H atoms were positioned geometrically [C—H = 0.93 or 0.98 Å] and were refined using a riding model, with *U*_iso_(H) = 1.2*U*_eq_(C). The dibromo substituted bromophenyl ring disordered over two sites with a refined occupany ratio of 0.756 (5):0.244 (5).

### Crystal data

**Table:**

C_17_H_11_Br_3_N_2_O_3_	*F*(000) = 2048
*M**_r_* = 531.01	*D*_x_ = 1.945 Mg m^−^^3^
Monoclinic, *C*2/*c*	Mo *K*α radiation, λ = 0.71073 Å
Hall symbol: -C 2yc	Cell parameters from 9996 reflections
*a* = 29.0105 (16) Å	θ = 2.4–32.1°
*b* = 7.2271 (4) Å	µ = 6.69 mm^−^^1^
*c* = 17.7209 (9) Å	*T* = 100 K
β = 102.591 (2)°	Block, colourless
*V* = 3626.0 (3) Å^3^	0.41 × 0.17 × 0.12 mm
*Z* = 8	

### Data collection

**Table:**

Bruker APEXII DUO CCD diffractometer	6547 independent reflections
Radiation source: fine-focus sealed tube	5223 reflections with *I* > 2σ(*I*)
graphite	*R*_int_ = 0.040
φ and ω scans	θ_max_ = 32.5°, θ_min_ = 2.4°
Absorption correction: multi-scan (*SADABS*; Bruker, 2009)	*h* = −43→43
*T*_min_ = 0.169, *T*_max_ = 0.503	*k* = −10→10
44623 measured reflections	*l* = −26→26

### Refinement

**Table:**

Refinement on *F*^2^	Primary atom site location: structure-invariant direct methods
Least-squares matrix: full	Secondary atom site location: difference Fourier map
*R*[*F*^2^ > 2σ(*F*^2^)] = 0.027	Hydrogen site location: inferred from neighbouring sites
*wR*(*F*^2^) = 0.099	H-atom parameters constrained
*S* = 1.04	*w* = 1/[σ^2^(*F*_o_^2^) + (0.0647*P*)^2^] where *P* = (*F*_o_^2^ + 2*F*_c_^2^)/3
6547 reflections	(Δ/σ)_max_ = 0.002
277 parameters	Δρ_max_ = 0.71 e Å^−^^3^
3 restraints	Δρ_min_ = −0.93 e Å^−^^3^

### Special details

**Table:**

Experimental. The crystal was placed in the cold stream of an Oxford Cryosystems Cobra open-flow nitrogen cryostat (Cosier & Glazer, 1986) operating at 100.0 (1) K.
Geometry. All s.u.'s (except the s.u. in the dihedral angle between two l.s. planes) are estimated using the full covariance matrix. The cell s.u.'s are taken into account individually in the estimation of s.u.'s in distances, angles and torsion angles; correlations between s.u.'s in cell parameters are only used when they are defined by crystal symmetry. An approximate (isotropic) treatment of cell s.u.'s is used for estimating s.u.'s involving l.s. planes.
Refinement. Refinement of F^2^ against ALL reflections. The weighted R-factor wR and goodness of fit S are based on F^2^, conventional R-factors R are based on F, with F set to zero for negative F^2^. The threshold expression of F^2^ > 2σ(F^2^) is used only for calculating R-factors(gt) etc. and is not relevant to the choice of reflections for refinement. R-factors based on F^2^ are statistically about twice as large as those based on F, and R- factors based on ALL data will be even larger.

### Fractional atomic coordinates and isotropic or equivalent isotropic displacement parameters (Å^2^)

**Table:**

	*x*	*y*	*z*	*U*_iso_*/*U*_eq_	Occ. (<1)
Br1A	0.18065 (5)	0.8649 (3)	0.50111 (5)	0.0454 (3)	0.756 (5)
Br2A	0.05273 (10)	0.6884 (4)	0.31021 (14)	0.0294 (3)	0.756 (5)
Br1B	0.0558 (3)	0.7183 (13)	0.3102 (5)	0.0306 (10)	0.244 (5)
Br2B	0.18785 (8)	0.8021 (5)	0.50059 (16)	0.0347 (4)	0.244 (5)
Br3	0.025545 (10)	0.93718 (3)	0.637488 (15)	0.04917 (9)	
O1	0.13831 (6)	1.30479 (19)	0.24590 (9)	0.0342 (3)	
O2	0.10960 (7)	1.2493 (2)	0.35316 (11)	0.0456 (4)	
O3	0.17640 (5)	0.7134 (2)	0.32498 (9)	0.0329 (3)	
N1	0.16750 (5)	1.0430 (2)	0.22747 (8)	0.0231 (3)	
N2	0.16194 (6)	1.2094 (2)	0.19957 (10)	0.0295 (3)	
C1	0.16918 (7)	0.7495 (3)	0.15957 (12)	0.0312 (4)	
H1A	0.1397	0.7196	0.1689	0.037*	
C2	0.19205 (8)	0.6312 (3)	0.11787 (14)	0.0388 (5)	
H2A	0.1782	0.5190	0.0998	0.047*	
C3	0.23549 (8)	0.6801 (3)	0.10321 (13)	0.0384 (5)	
H3A	0.2504	0.6015	0.0745	0.046*	
C4	0.25712 (7)	0.8466 (3)	0.13125 (12)	0.0339 (4)	
H4A	0.2862	0.8783	0.1211	0.041*	
C5	0.23519 (6)	0.9649 (3)	0.17425 (11)	0.0278 (3)	
H5A	0.2494	1.0753	0.1940	0.033*	
C6	0.19154 (6)	0.9134 (2)	0.18684 (10)	0.0231 (3)	
C7	0.12899 (8)	1.1883 (3)	0.30537 (13)	0.0322 (4)	
C8	0.14869 (6)	1.0146 (3)	0.29097 (11)	0.0262 (3)	
C9	0.15335 (7)	0.8457 (3)	0.33621 (12)	0.0289 (4)	
C10A	0.12988 (9)	0.8495 (3)	0.40719 (15)	0.0244 (5)	0.756 (5)
H10A	0.1088	0.9566	0.4041	0.029*	0.756 (5)
C11A	0.10229 (8)	0.6727 (3)	0.40790 (13)	0.0218 (5)	0.756 (5)
H11B	0.1231	0.5680	0.4038	0.026*	0.756 (5)
C12A	0.08001 (10)	0.6399 (3)	0.47605 (16)	0.0222 (5)	0.756 (5)
C13A	0.06496 (12)	0.7851 (4)	0.51665 (19)	0.0248 (5)	0.756 (5)
H13A	0.0679	0.9067	0.5012	0.030*	0.756 (5)
C14A	0.0457 (2)	0.7482 (9)	0.5796 (3)	0.0265 (10)	0.756 (5)
C15A	0.0414 (2)	0.5602 (13)	0.6012 (4)	0.0318 (16)	0.756 (5)
H15A	0.0292	0.5348	0.6445	0.038*	0.756 (5)
C16A	0.05400 (18)	0.4211 (8)	0.5623 (3)	0.0292 (11)	0.756 (5)
H16A	0.0496	0.2999	0.5768	0.035*	0.756 (5)
C17A	0.07444 (16)	0.4577 (5)	0.4975 (3)	0.0284 (7)	0.756 (5)
H17A	0.0839	0.3607	0.4699	0.034*	0.756 (5)
C10B	0.1119 (3)	0.8207 (10)	0.3781 (5)	0.0198 (14)*	0.244 (5)
H10B	0.1041	0.9404	0.3981	0.024*	0.244 (5)
C11B	0.1295 (2)	0.6921 (9)	0.4440 (4)	0.0204 (15)*	0.244 (5)
H11A	0.1376	0.5748	0.4223	0.024*	0.244 (5)
C12B	0.0973 (3)	0.6488 (10)	0.4974 (4)	0.0187 (15)*	0.244 (5)
C13B	0.0797 (3)	0.7882 (14)	0.5365 (5)	0.025 (2)*	0.244 (5)
H13B	0.0864	0.9126	0.5307	0.030*	0.244 (5)
C14B	0.0502 (7)	0.726 (3)	0.5867 (12)	0.026 (4)*	0.244 (5)
C15B	0.0365 (7)	0.569 (3)	0.6037 (12)	0.017 (3)*	0.244 (5)
H15B	0.0170	0.5432	0.6377	0.020*	0.244 (5)
C16B	0.0593 (5)	0.426 (3)	0.5548 (9)	0.030 (4)*	0.244 (5)
H16B	0.0527	0.3017	0.5602	0.036*	0.244 (5)
C17B	0.0849 (4)	0.466 (2)	0.5104 (7)	0.022 (3)*	0.244 (5)
H17B	0.0966	0.3705	0.4844	0.027*	0.244 (5)

### Atomic displacement parameters (Å^2^)

**Table:**

	*U*^11^	*U*^22^	*U*^33^	*U*^12^	*U*^13^	*U*^23^
Br1A	0.0404 (4)	0.0737 (6)	0.02262 (17)	−0.0314 (4)	0.0079 (2)	−0.0057 (3)
Br2A	0.0239 (4)	0.0422 (8)	0.0217 (2)	−0.0043 (4)	0.0041 (2)	−0.0007 (3)
Br1B	0.0210 (9)	0.041 (2)	0.0279 (8)	−0.0050 (12)	0.0014 (6)	0.0034 (10)
Br2B	0.0254 (5)	0.0484 (11)	0.0291 (5)	−0.0107 (6)	0.0032 (4)	0.0032 (7)
Br3	0.07536 (19)	0.03488 (13)	0.05149 (15)	0.00417 (10)	0.04489 (14)	−0.00002 (9)
O1	0.0490 (9)	0.0236 (7)	0.0339 (8)	0.0021 (6)	0.0171 (7)	0.0043 (5)
O2	0.0697 (12)	0.0275 (7)	0.0513 (10)	0.0098 (7)	0.0387 (9)	0.0009 (7)
O3	0.0340 (7)	0.0304 (7)	0.0396 (8)	0.0090 (5)	0.0199 (6)	0.0083 (6)
N1	0.0252 (7)	0.0232 (7)	0.0221 (7)	−0.0027 (5)	0.0079 (5)	0.0004 (5)
N2	0.0386 (9)	0.0250 (8)	0.0275 (8)	−0.0013 (6)	0.0125 (6)	0.0032 (6)
C1	0.0294 (9)	0.0327 (9)	0.0333 (10)	−0.0048 (7)	0.0112 (7)	−0.0068 (8)
C2	0.0440 (11)	0.0342 (11)	0.0404 (11)	−0.0014 (9)	0.0142 (9)	−0.0093 (9)
C3	0.0410 (11)	0.0443 (12)	0.0337 (11)	0.0127 (9)	0.0167 (9)	−0.0035 (9)
C4	0.0268 (8)	0.0454 (12)	0.0325 (10)	0.0052 (8)	0.0132 (7)	0.0060 (8)
C5	0.0245 (8)	0.0345 (10)	0.0258 (8)	−0.0034 (7)	0.0084 (6)	0.0033 (7)
C6	0.0238 (7)	0.0257 (8)	0.0214 (7)	−0.0010 (6)	0.0086 (6)	−0.0011 (6)
C7	0.0438 (11)	0.0204 (8)	0.0373 (10)	0.0002 (7)	0.0197 (9)	0.0032 (7)
C8	0.0299 (8)	0.0230 (8)	0.0297 (8)	−0.0002 (6)	0.0156 (7)	0.0017 (6)
C9	0.0324 (9)	0.0257 (9)	0.0340 (10)	0.0018 (7)	0.0193 (8)	0.0040 (7)
C10A	0.0270 (12)	0.0253 (11)	0.0228 (11)	−0.0028 (8)	0.0096 (10)	−0.0011 (8)
C11A	0.0226 (10)	0.0221 (10)	0.0219 (10)	0.0008 (7)	0.0074 (8)	0.0022 (7)
C12A	0.0224 (12)	0.0236 (11)	0.0219 (11)	−0.0012 (8)	0.0076 (10)	0.0035 (8)
C13A	0.0290 (14)	0.0215 (12)	0.0270 (13)	−0.0004 (9)	0.0128 (12)	0.0039 (9)
C14A	0.0311 (19)	0.025 (2)	0.0270 (19)	0.0044 (15)	0.0141 (11)	0.0063 (14)
C15A	0.030 (2)	0.040 (3)	0.0304 (19)	0.0011 (17)	0.0159 (16)	0.0112 (13)
C16A	0.0343 (18)	0.0251 (17)	0.0322 (18)	−0.0033 (12)	0.0161 (16)	0.0112 (11)
C17A	0.0315 (18)	0.0239 (14)	0.0297 (17)	−0.0019 (13)	0.0065 (15)	0.0032 (12)

### Geometric parameters (Å, °)

**Table:**

Br1A—C10A	1.971 (3)	C10A—C11A	1.509 (3)
Br2A—C11A	1.998 (4)	C10A—H10A	0.9800
Br1B—C10B	1.947 (12)	C11A—C12A	1.508 (3)
Br2B—C11B	1.940 (8)	C11A—H11B	0.9800
Br3—C14A	1.876 (7)	C12A—C17A	1.390 (5)
Br3—C14B	1.98 (2)	C12A—C13A	1.395 (4)
O1—N2	1.366 (2)	C13A—C14A	1.379 (7)
O1—C7	1.420 (2)	C13A—H13A	0.9300
O2—C7	1.198 (2)	C14A—C15A	1.424 (12)
O3—C9	1.208 (2)	C15A—C16A	1.316 (11)
N1—N2	1.297 (2)	C15A—H15A	0.9300
N1—C8	1.369 (2)	C16A—C17A	1.427 (7)
N1—C6	1.450 (2)	C16A—H16A	0.9300
C1—C6	1.386 (3)	C17A—H17A	0.9300
C1—C2	1.390 (3)	C10B—C11B	1.493 (10)
C1—H1A	0.9300	C10B—H10B	0.9800
C2—C3	1.387 (3)	C11B—C12B	1.502 (10)
C2—H2A	0.9300	C11B—H11A	0.9800
C3—C4	1.397 (3)	C12B—C13B	1.383 (12)
C3—H3A	0.9300	C12B—C17B	1.404 (16)
C4—C5	1.388 (3)	C13B—C14B	1.44 (3)
C4—H4A	0.9300	C13B—H13B	0.9300
C5—C6	1.384 (2)	C14B—C15B	1.26 (4)
C5—H5A	0.9300	C15B—C16B	1.58 (3)
C7—C8	1.425 (3)	C15B—H15B	0.9300
C8—C9	1.450 (3)	C16B—C17B	1.23 (2)
C9—C10B	1.555 (7)	C16B—H16B	0.9300
C9—C10A	1.556 (3)	C17B—H17B	0.9300
			
C14A—Br3—C14B	6.0 (6)	C17A—C12A—C13A	120.2 (3)
N2—O1—C7	110.39 (14)	C17A—C12A—C11A	117.6 (3)
N2—N1—C8	114.43 (15)	C13A—C12A—C11A	122.2 (2)
N2—N1—C6	116.31 (14)	C14A—C13A—C12A	120.0 (3)
C8—N1—C6	129.25 (15)	C14A—C13A—H13A	120.0
N1—N2—O1	106.03 (14)	C12A—C13A—H13A	120.0
C6—C1—C2	118.03 (18)	C13A—C14A—C15A	118.5 (6)
C6—C1—H1A	121.0	C13A—C14A—Br3	122.1 (4)
C2—C1—H1A	121.0	C15A—C14A—Br3	119.4 (5)
C3—C2—C1	120.1 (2)	C16A—C15A—C14A	122.5 (7)
C3—C2—H2A	120.0	C16A—C15A—H15A	118.8
C1—C2—H2A	120.0	C14A—C15A—H15A	118.8
C2—C3—C4	120.60 (19)	C15A—C16A—C17A	119.5 (6)
C2—C3—H3A	119.7	C15A—C16A—H16A	120.2
C4—C3—H3A	119.7	C17A—C16A—H16A	120.2
C5—C4—C3	120.10 (18)	C12A—C17A—C16A	119.3 (4)
C5—C4—H4A	120.0	C12A—C17A—H17A	120.4
C3—C4—H4A	120.0	C16A—C17A—H17A	120.4
C6—C5—C4	117.87 (18)	C11B—C10B—C9	106.1 (5)
C6—C5—H5A	121.1	C11B—C10B—Br1B	110.1 (6)
C4—C5—H5A	121.1	C9—C10B—Br1B	112.3 (5)
C5—C6—C1	123.31 (17)	C11B—C10B—H10B	109.4
C5—C6—N1	117.53 (16)	C9—C10B—H10B	109.4
C1—C6—N1	119.09 (15)	Br1B—C10B—H10B	109.4
O2—C7—O1	120.04 (17)	C10B—C11B—C12B	117.9 (6)
O2—C7—C8	136.00 (19)	C10B—C11B—Br2B	105.1 (5)
O1—C7—C8	103.93 (16)	C12B—C11B—Br2B	110.6 (5)
N1—C8—C7	105.22 (16)	C10B—C11B—H11A	107.6
N1—C8—C9	125.07 (16)	C12B—C11B—H11A	107.6
C7—C8—C9	129.42 (17)	Br2B—C11B—H11A	107.6
O3—C9—C8	124.29 (17)	C13B—C12B—C17B	118.1 (9)
O3—C9—C10B	120.0 (3)	C13B—C12B—C11B	120.9 (7)
C8—C9—C10B	111.9 (3)	C17B—C12B—C11B	121.0 (8)
O3—C9—C10A	120.18 (18)	C12B—C13B—C14B	114.6 (12)
C8—C9—C10A	115.32 (17)	C12B—C13B—H13B	122.7
C10B—C9—C10A	25.3 (3)	C14B—C13B—H13B	122.7
C11A—C10A—C9	108.56 (19)	C15B—C14B—C13B	134 (2)
C11A—C10A—Br1A	109.92 (18)	C15B—C14B—Br3	115 (2)
C9—C10A—Br1A	107.79 (17)	C13B—C14B—Br3	111.2 (14)
C11A—C10A—H10A	110.2	C14B—C15B—C16B	105 (2)
C9—C10A—H10A	110.2	C14B—C15B—H15B	127.3
Br1A—C10A—H10A	110.2	C16B—C15B—H15B	127.3
C12A—C11A—C10A	117.4 (2)	C17B—C16B—C15B	126 (2)
C12A—C11A—Br2A	110.47 (18)	C17B—C16B—H16B	117.1
C10A—C11A—Br2A	103.15 (19)	C15B—C16B—H16B	117.1
C12A—C11A—H11B	108.5	C16B—C17B—C12B	122.3 (16)
C10A—C11A—H11B	108.5	C16B—C17B—H17B	118.9
Br2A—C11A—H11B	108.5	C12B—C17B—H17B	118.9
			
C8—N1—N2—O1	0.5 (2)	Br2A—C11A—C12A—C17A	91.6 (3)
C6—N1—N2—O1	179.26 (14)	C10A—C11A—C12A—C13A	29.9 (4)
C7—O1—N2—N1	−0.1 (2)	Br2A—C11A—C12A—C13A	−87.9 (3)
C6—C1—C2—C3	−1.4 (3)	C17A—C12A—C13A—C14A	1.8 (6)
C1—C2—C3—C4	1.2 (4)	C11A—C12A—C13A—C14A	−178.6 (4)
C2—C3—C4—C5	0.0 (3)	C12A—C13A—C14A—C15A	−0.5 (6)
C3—C4—C5—C6	−1.0 (3)	C12A—C13A—C14A—Br3	179.2 (3)
C4—C5—C6—C1	0.8 (3)	C14B—Br3—C14A—C13A	−133 (8)
C4—C5—C6—N1	−176.10 (17)	C14B—Br3—C14A—C15A	46 (8)
C2—C1—C6—C5	0.3 (3)	C13A—C14A—C15A—C16A	−1.7 (7)
C2—C1—C6—N1	177.24 (19)	Br3—C14A—C15A—C16A	178.7 (4)
N2—N1—C6—C5	53.4 (2)	C14A—C15A—C16A—C17A	2.4 (7)
C8—N1—C6—C5	−128.0 (2)	C13A—C12A—C17A—C16A	−1.2 (5)
N2—N1—C6—C1	−123.66 (19)	C11A—C12A—C17A—C16A	179.3 (3)
C8—N1—C6—C1	54.9 (3)	C15A—C16A—C17A—C12A	−1.0 (6)
N2—O1—C7—O2	177.9 (2)	O3—C9—C10B—C11B	42.9 (7)
N2—O1—C7—C8	−0.3 (2)	C8—C9—C10B—C11B	−158.2 (4)
N2—N1—C8—C7	−0.7 (2)	C10A—C9—C10B—C11B	−55.0 (6)
C6—N1—C8—C7	−179.25 (18)	O3—C9—C10B—Br1B	−77.5 (5)
N2—N1—C8—C9	−175.01 (18)	C8—C9—C10B—Br1B	81.5 (5)
C6—N1—C8—C9	6.4 (3)	C10A—C9—C10B—Br1B	−175.3 (10)
O2—C7—C8—N1	−177.2 (3)	C9—C10B—C11B—C12B	177.3 (6)
O1—C7—C8—N1	0.6 (2)	Br1B—C10B—C11B—C12B	−60.9 (8)
O2—C7—C8—C9	−3.2 (4)	C9—C10B—C11B—Br2B	53.5 (6)
O1—C7—C8—C9	174.53 (19)	Br1B—C10B—C11B—Br2B	175.3 (4)
N1—C8—C9—O3	3.7 (3)	C10B—C11B—C12B—C13B	−57.9 (9)
C7—C8—C9—O3	−169.2 (2)	Br2B—C11B—C12B—C13B	63.1 (7)
N1—C8—C9—C10B	−154.2 (4)	C10B—C11B—C12B—C17B	123.5 (8)
C7—C8—C9—C10B	32.9 (5)	Br2B—C11B—C12B—C17B	−115.5 (6)
N1—C8—C9—C10A	178.39 (19)	C17B—C12B—C13B—C14B	0.0 (3)
C7—C8—C9—C10A	5.5 (3)	C11B—C12B—C13B—C14B	−178.7 (8)
O3—C9—C10A—C11A	−51.0 (3)	C12B—C13B—C14B—C15B	0.1 (4)
C8—C9—C10A—C11A	134.0 (2)	C12B—C13B—C14B—Br3	−178.5 (7)
C10B—C9—C10A—C11A	46.1 (6)	C14A—Br3—C14B—C15B	−120 (8)
O3—C9—C10A—Br1A	68.0 (2)	C14A—Br3—C14B—C13B	59 (8)
C8—C9—C10A—Br1A	−106.97 (19)	C13B—C14B—C15B—C16B	−0.1 (6)
C10B—C9—C10A—Br1A	165.1 (7)	Br3—C14B—C15B—C16B	178.4 (8)
C9—C10A—C11A—C12A	174.8 (2)	C14B—C15B—C16B—C17B	0.2 (8)
Br1A—C10A—C11A—C12A	57.2 (2)	C15B—C16B—C17B—C12B	−0.1 (9)
C9—C10A—C11A—Br2A	−63.4 (2)	C13B—C12B—C17B—C16B	0.0 (6)
Br1A—C10A—C11A—Br2A	178.89 (13)	C11B—C12B—C17B—C16B	178.7 (9)
C10A—C11A—C12A—C17A	−150.5 (3)		

### Hydrogen-bond geometry (Å, °)

**Table:**

*D*—H···*A*	*D*—H	H···*A*	*D*···*A*	*D*—H···*A*
C5—H5A···O3^i^	0.93	2.46	3.129 (2)	129
C10A—H10A···O2	0.98	2.30	3.060 (3)	133
C11A—H11B···O2^ii^	0.98	2.47	3.231 (3)	134
C17A—H17A···O2^ii^	0.93	2.48	3.315 (5)	149

Symmetry codes: (i) *x*, −*y*, *z*−1/2; (ii) *x*, *y*−1, *z*.
